# A comparative study of the impact of maternal immune activation and valproic acid on autism-like behavior in rats

**DOI:** 10.1515/tnsci-2025-0402

**Published:** 2026-08-24

**Authors:** Maha K. Alaskar, Mona Alonazi, Abir Ben Bacha, Abdulaziz M. Alamri, Sameera Abuaish, Hisham S. Aloudah, Mohammed Fahad Alahmed, Ahmad Tayseer AlMnaizel, Afaf K. El-Ansary

**Affiliations:** Department of Biochemistry, College of Sciences, King Saud University, Riyadh, Saudi Arabia; Department of Chemistry, College of Science, Imam Mohammad Ibn Saud Islamic University (IMSIU), Riyadh, Saudi Arabia; Department of Basic Sciences, College of Medicine, Princess Nourah Bint Abdulrahman University, Riyadh, Saudi Arabia; Prince Naif for Health Research Center, King Saud University, Riyadh, Saudi Arabia; Research Office, John Hopkins Aramco Healthcare, Dhahran, Saudi Arabia; Autism Research and Treatment Center, King Saud University, Riyadh, Saudi Arabia

**Keywords:** autism, Valproic Acid (VPA), maternal immune activation (MIA), lipopolysaccharide (LPS), social interaction, artichoke

## Abstract

**Objectives:**

The present study aimed to evaluate the behavioral consequences of maternal immune activation (MIA) induced by lipopolysaccharide (LPS), in comparison to valproic acid (VPA), a widely used and reliable inducer of autism-like features in rats when administered during prenatal development. At the behavioral level, we further assessed the potential ameliorative effects of a standardized polyphenol-rich extract derived from the leaves of *Cynara cardunculus* L. (artichoke), used as a prebiotic – either independently or in combination with a probiotic mixture and/or omega-3 (ω3) fatty acids.

**Methods:**

Wistar albino rats prenatally exposed to LPS or VPA were evaluated for social behavior using the three-chamber social interaction test. Beginning at 7 days of age, male rat pups received specific doses of the artichoke as prebiotic (AR), probiotics (Pro), and ω3 supplements, depending on group assignment. A total of 54 neonatal male rats were divided into nine experimental groups: (1) Control group: received normal saline; (2) VPA-0 group: prenatally exposed to VPA only; (3) VPA-AR group: prenatally exposed to VPA, postnatally treated with AR; (4) VPA-AR.Pro group: prenatally exposed to VPA, postnatally treated with AR + Pro; (5) VPA.AR-AR group: received AR during gestation (protectively), and postnatal AR after VPA exposure; (6) LPS-0 group: prenatally exposed to LPS only; (7) LPS-AR group: prenatally exposed to LPS, postnatally treated with AR; (8) LPS-AR.Pro.ω3 group: prenatally exposed to LPS, postnatally treated with AR + Pro + ω3; (9) LPS.AR-AR group: received AR during gestation (protectively), and postnatal AR after LPS exposure.

**Results:**

LPS and prenatal VPA exposure significantly impair social interactions and promoting stereotypic-like behaviors. Notably, both pre- and postnatal administration of artichoke extract demonstrated significant potential in improving these behavioral deficits. MIA and prenatal VPA exposure have detrimental effects on neurobehavioral development, particularly impairing social interactions and promoting stereotypic-like behaviors consistent with ASD.

**Conclusions:**

These results provide evidence for the beneficial role of artichoke-derived prebiotics, alone or in combination with probiotics and ω3, in improving ASD-like symptoms in rodent models of autism.

## Introduction

Autism Spectrum Disorder (ASD) is characterized by a wide range of symptoms and severity levels, thus the term “spectrum”. Common features include difficulties with social interaction and communication, as well as repetitive behaviors and restricted interests [1], [2], [3]. In 2025, autism is estimated to affect approximately 1 in 31 children in the United States according to the Centers for Disease Control and Prevention’s [4]. Globally, the World Health Organization estimates that about 1 in 100 children are diagnosed with autism [5].

Animal models are crucial in autism research to understand the complex biological and behavioral aspects of autism in a controlled environment, which is often impossible with human subjects. These models, particularly rodents, offer a platform to investigate the genetic, environmental, and epigenetic factors contributing to autism, as well as to test potential therapeutic interventions [6], 7]. Valproic acid (VPA), or 2-propylpentanoic acid, is a short-chained fatty acid widely used as an antiepileptic drug and has been utilized to model autism in rodents. VPA exposure in pregnant rats causes offspring to exhibit core ASD features such as impaired social interaction, increased repetitive behaviors, and altered sensory processing, making it an excellent model for studying the biological mechanisms of ASD and testing potential treatments [8], [9], [10], [11].

Numerous studies have linked maternal infections, autoimmune conditions, and high Body Mass Index (BMI) during pregnancy to an increased risk of ASD in offspring, an association mediated by Maternal Immune Activation (MIA). [12], [13], [14], [15]. MIA is a condition where the mother’s immune system is activated during pregnancy, either by infection (bacterial or viral) or other inflammatory stimuli. This activation can lead to the release of inflammatory molecules (cytokines), which can cross the placenta and affect the developing fetus [12], 15], 16].

Lipopolysaccharide (LPS), is a molecule found in the outer membrane of Gram-negative bacteria and a potent activator of the innate immune system, triggering the release of cytokines [17]. MIA by LPS refers to a process where exposure of a pregnant mother to LPS triggers an immune response in the mother. This immune response can have lasting effects on the developing offspring, potentially leading to neurodevelopmental issues. LPS binds to Toll-like receptor 4 (TLR4) on maternal immune cells (macrophages, monocytes, dendritic cells), initiating a strong inflammatory cascade and leads to disrupting fetal brain development and potentially increase the risk of ASD in offspring [16], [18], [19], [20].

Moreover, offspring exposed to LPS-induced MIA often exhibit a range of behavioral and neurobiological deficits that resemble symptoms of neurodevelopmental disorders such as social interaction deficits, repetitive behaviors (increased grooming, stereotypies), communication impairments, cognitive deficits, and anxiety-like behaviors [12], [13], [14].

Despite extensive preclinical and clinical research, there are currently no effective preventive or therapeutic strategies to mitigate the impact of maternal inflammation on fetal brain development. With no cure for ASD and it’s continuing rise, there is growing interest in dietary interventions. However, there is no consensus on the optimal nutritional approach. The potential of specific dietary compositions to regulate or alleviate ASD symptoms has been highlighted in recent reviews [21], 22].

Probiotics, which are living bacteria that provide health advantages to the host, and prebiotics, which are nondigestible compounds used exclusively by beneficial gut microbiota to boost host health, are two of the most promising dietary strategies [23], [24], [25].

Artichoke-derived inulin possesses an exceptionally high degree of polymerization, ensuring prolonged colonic persistence and potent prebiotic efficacy. By selectively modulating gut microbiota, inulin increases short-chain fatty acid (SCFA) production, which acidifies the colon to favor beneficial taxa (*Bifidobacterium*, *Lactobacillus*) and suppress pathogens [26], 27]. These microbial shifts further fortify host immunity [28], 29]. Additionally, artichoke polyphenols (e.g., dicaffeoylquinic acids, flavonoids) likely synergize with inulin to optimize gastrointestinal metabolic and oxidative health [30], [31], [32]. Artichoke extracts can act as antioxidants and anti-inflammatories, which may be beneficial in protecting against various insults during pregnancy. In 2032, a study done by Alsubaiei and collaborators, has shown that supplementation with yogurt, artichoke (as a prebiotic source), or specific probiotic strains (e.g., *Lacticaseibacillus rhamnosus* GG) can improve biochemical markers of oxidative stress (increase GSH, GPx) and reduce neuroinflammation (decrease IL-6, TNF-α) in the brains of propionic acid-treated rats, correlating with behavioral improvements [33].

Despite these robust gut-modulating properties, their application in autism is largely uncharacterized. Consequently, this study evaluates the impact of artichoke prebiotics and synbiotic formulations on behavioral deficits in LPS- and VPA-induced autism rat models.

This study builds upon previous research and being a part of our KSU research team into the use of artichoke as a therapeutic intervention to improve behavioral consequences of MIA [33], 34].

## Materials and methods

### Animal model and ethics statement

All experimental procedures were carried out in the Center for Experimental Surgery and Animals Lab, Prince Naif Health Research Center (PNHRC), King Khalid University Hospital (KKUH), Riyadh, Saudia Arabia. The experimental procedure was pre-approved by the Ethical Committee of Bioethics at King Saud University (KSU), Ref Number: SE-24-33.

All animals hosted in polypropylene cages in an environmentally controlled clean air room, with a temperature of (25 °C ± 1 °C) 12 h light/12 h dark cycle and a relative humidity of 50 ± 5 %.

**Figure 1:**
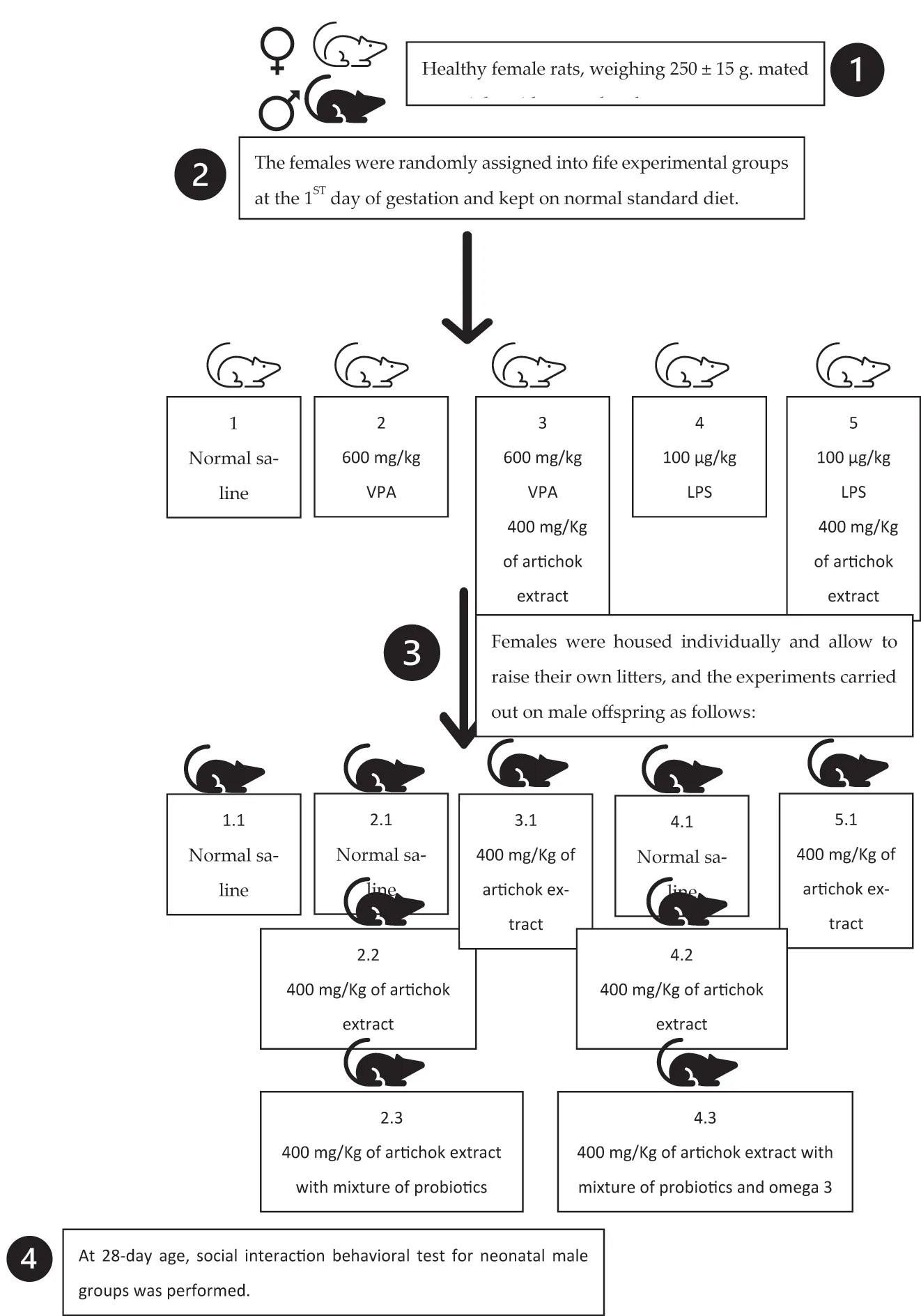
Diagrammatic scheme of the animal experiments.

Sixteen healthy female Wistar albino rats (8 ± 1 weeks old, weighting 250 ± 15 g), were mated overnight with male rats in the proportion of 2:1, and the day spermatozoa were detected in the vaginal smear, was designated as the first day of gestation (Figure 1).

The pregnant females were randomly assigned into fife experimental groups as follows:

Group.1: 2 Control females were kept on normal standard diet and injected with physiological saline.

Group.2: 5 Females were kept on normal standard diet and received a single intraperitoneal (IP) injection of 600 mg/kg sodium valproate on day 12.5 after conception.

Group.3: 2 Females were kept on normal standard diet and received oral administration of 400 mg/Kg of artichoke daily from the 1st day of gestation until the date of birth. Also, they received a single IP injection of 600 mg/kg VPA on day 12.5 after conception.

Group.4: 5 Females were kept on normal standard diet and received a single IP injection of 100 µg/kg LPS on day 9.5 after conception.

Group.5: 2 Females were kept on normal standard diet and received oral administration of 400 mg/Kg of artichoke daily from the 1st day of gestation until the date of birth. Also, they received a single IP injection of 100 µg/kg LPS on day 9.5 after conception.

Females were housed individually and allowed to raise their own litters, and the experiments were carried out on male offspring as follows:

Group.1.1: 6 male puppies received normal saline orally after day 7 of birth (control).

Group.2.1: 6 male puppies were used as valproate-exposed rats (VPA-0).

Group.2.2: 6 male puppies were received 400 mg/Kg of artichoke orally after day 7 of birth (VPA-AR).

Group.2.3: 6 male puppies were received a mix of 400 mg/Kg of artichoke and mixture of probiotics after day 7 of birth (VPA-AR.Pro).

Group.3.1: 6 male puppies were received 400 mg/Kg of artichoke orally after day 7 of birth (VPA.AR-AR).

Group.4.1: 6 male puppies were used as LPS-exposed rats (LPS-0)

Group.4.2: 6 male puppies were received 400 mg/Kg of artichoke orally after day 7 of birth (LPS-AR).

Group.4.3: 6 male puppies were received a mix of 400 mg/Kg of artichoke and mixture of probiotics with omega −3 after day 7 of birth (LPS-AR.Pro.ω3).

Group.5.1: 6 male puppies were received 400 mg/Kg of artichoke orally after day 7 of birth (LPS.AR-AR).

At the end of the experiment, a three-chamber social test was performed with offspring beginning at 4 weeks of age.

### Drugs & dosage

#### Sodium valproate (VPA)

VPA salt was obtained from Sigma-Aldrich (Catalog No. P4543). It was dissolved in normal saline (0.9 % NaCl) at a concentration of 250 mg/ml as described by Schneideret al., (2005). Exposure to VPA around embryonic day 12 (E12) is associated with the highest susceptibility for ASD development [35]. While the commonly used VPA dose in animal models of ASD is 600 mg/kg, it is important to note that clinical doses of VPA in humans range from approximately 3 to 55 mg/kg, corresponding to a plasma concentration of ∼100 μg/mL. Thus, the dose used in animal studies is typically 10–20 times higher than therapeutic levels in humans [8], 36].

#### Lipopolysaccharide (LPS)

LPS (from *Escherichia coli*, serotype 055: B5, Solarbio, Beijing, China) was dissolved in saline solution at a concentration of 100 µg/kg and administered to pregnant dams (I.P. in a volume of 2 mL/kg at GD 9.5) in order to induce some autistic-like behavioral changes in the rat offspring as previously reported [37], 38].

#### Extract from cynara scolymus L. (Artichoke)

The shade-dried powdered artichoke (1 Kg) was extracted with 96 % ethanol (4 × 1 L), using Soxhlet equipment for 3 h at 80 °C. All the extracts were combined and filtered through the Whatman paper No 1. The filtrate was then freed from the solvent under reduced pressure at ± 40 °C temperature using rotavapor, resulting in a dark green extract (101.7 g). The extract was placed in the hood to ensure complete ethanol evaporation. The final dry ethanolic extract was stored at 4 °C until further use. Artichoke extract was diluted in distilled water at a final concentration of 400 mg/kg. The dose of artichoke used in this study was determined based on previous studies [33], 34], 39].

#### Mixture of probiotics

Infant probiotics (LoveBug Nutrition, NY) obtained from iHerb, are a mixture of healthy bacteria, including *Bifidobacterium infants*, *Bifidobacterium lactis and Lactobacillus rhamnosus GG*, with the concentration of 1 billion CFU per 1.5 g [40].

#### Omega-3 polyunsaturated fatty acids (or omega-3 PUFAs, n-3 PUFAs)

Omega-3 Premium Fish Oil (from California Gold Nutrition, USA) obtained from iHerb, was orally given at a dose of 200 mg/kg body weight/day [41].

### Evaluation of social behavior

Social behavior, an important feature of ASD, was assessed using the three-chamber social test, a commonly used test to evaluate social impairments in animal models of autism [42]. A 120 cm × 40 cm × 40 cm clear rectangular plexiglass box divided into three chambers with walls and 15 cm × 15 cm doors with removable slides to allow the animals to pass through. The three-chamber box was cleaned with 70 % ethanol, dried with paper towels, and then let to air dry between trails. The animals were transferred to the testing room 1 h before the test for acclimatization. Animals were picked from the cage and placed individually in the center chamber and allowed to explore for 5 min while the two doorways of the box were open. Following this habituation period, an age, weigh and sex-matched stranger rat (conspecific) to the testing rats was placed in one of two perforated holding containers that were located on either side of the box. The subject rat was allowed to explore all three chambers freely for 10 min. The test was recorded using an HD camcorder. Videos were later analyzed to code behaviour using BORIS 7.9.16 software [43]. Social interaction time was quantified by measuring the focal animal’s orientation towards and investigating the containing the conspecific, evidenced by sniffing or rearing. Time spent in each chamber was analyzed, and the percentage of time spent in the social chamber (containing the conspecific rat) relative to the non-social chamber (containing the empty holding container) and the center chamber were calculated. Grooming was analyzed for total duration and the location where it was performed, comparing time spent in the stimulus-containing chamber vs. the empty chamber. Time spent immobile (bouts of no movements) was recorded. To assess activity, locomotion was analyzed for total duration.

### Statistical analysis

Behavioral data were analyzed using one-way ANOVA by GraphPad Prism 10. Ink, software to evaluate the effects of VPA and LPS on social interaction, grooming, immobility and locomotion in experimental following groups (n=6 per group): Control, VPA (valproic acid; autism model group), VPA-AR (artichoke treatment group), VPA-AR.PRO (artichoke and probiotic combination treatment group), VPA.AR-AR (artichoke protective treatment group), LPS (lipopolysaccharide; autism model group), LPS-AR (artichoke treatment group), LPS-AR.PRO.ω3 (artichoke, probiotic, and omega-3 combination treatment group), and LPS.AR-AR (artichoke protective treatment group). Tukey’s post hoc tests were used for pairwise comparisons, with p≤0.05 considered significant. Data are presented as mean +/− standard errors of the mean. Significant differences are indicated as follows: ****p≤0.0001, ***p≤0.001, *p≤0.01, p≤0.05.

## Results

**Figure 2:**
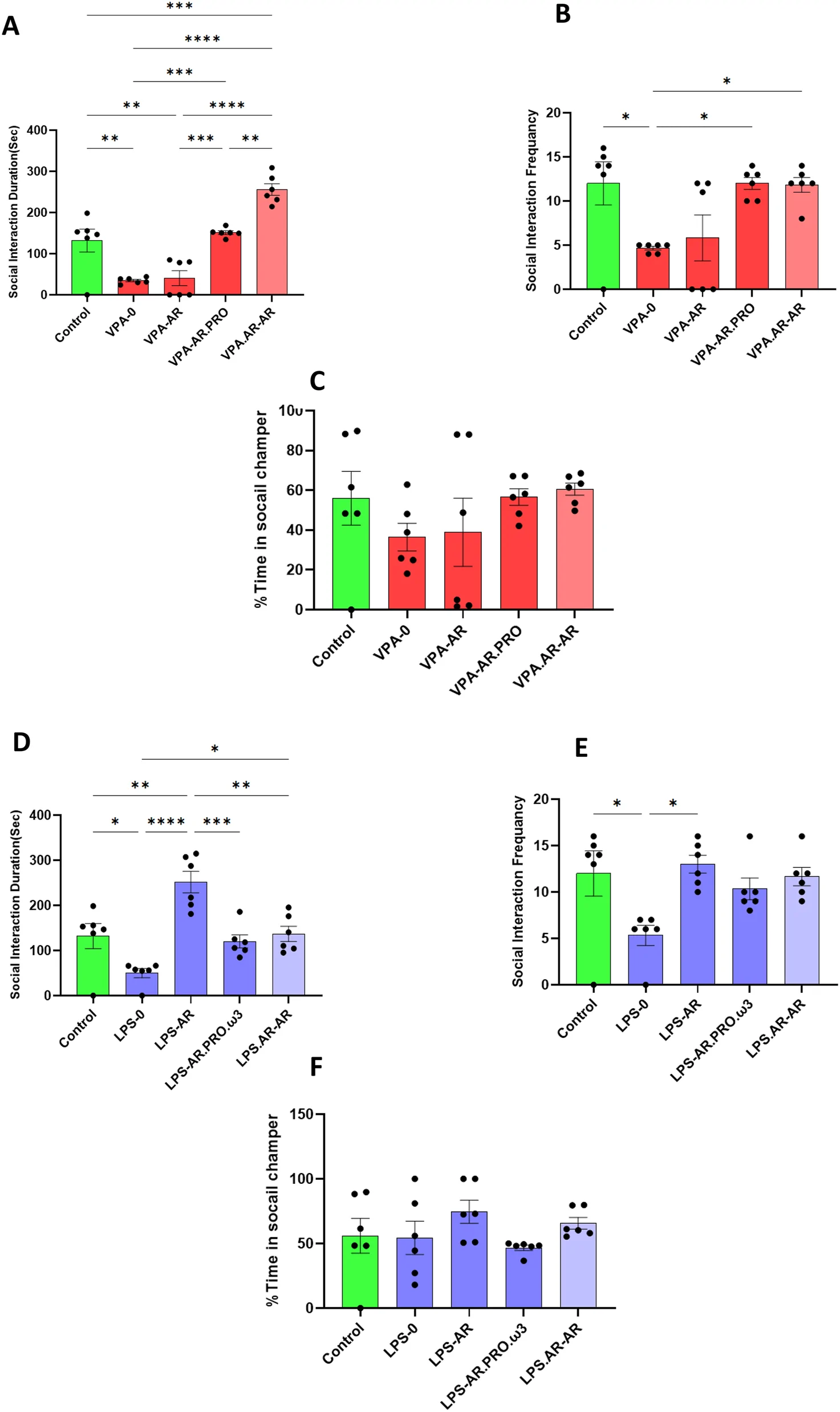
Social interaction duration and frequency in control and experimental groups. Saline (n=6), VPA (n=6; autism model), VPA-AR (n=6; artichoke treatment), VPA-AR.PRO (n=6; artichoke + probiotic), VPA-AR-AR (n=6; protective), LPS (n=6; autism model), LPS-AR (n=6; artichoke treatment), LPS-AR.PRO.ω3 (n=6; artichoke + probiotic + omega-3), and LPS-AR-AR (n=6; protective). Data are presented as mean ± SEM. One-way ANOVA, (**** p≤0.0001, *** p≤0.001, ** p≤0.01, * p≤0.05). A, social interaction total duration of VPA; B, social interaction frequency of VPA; C, percentage of time spent in the social chamber relative to total test duration of VPA; D, social interaction total duration of LPS; E, social interaction frequency of LPS and F, percentage of time spent in the social chamber relative to total test duration of LPS; VPA, valproic acid; AR, artichoke; Pro, probiotics; ω3, omega3; m LPS, lipopolysaccharide.

**Figure 3:**
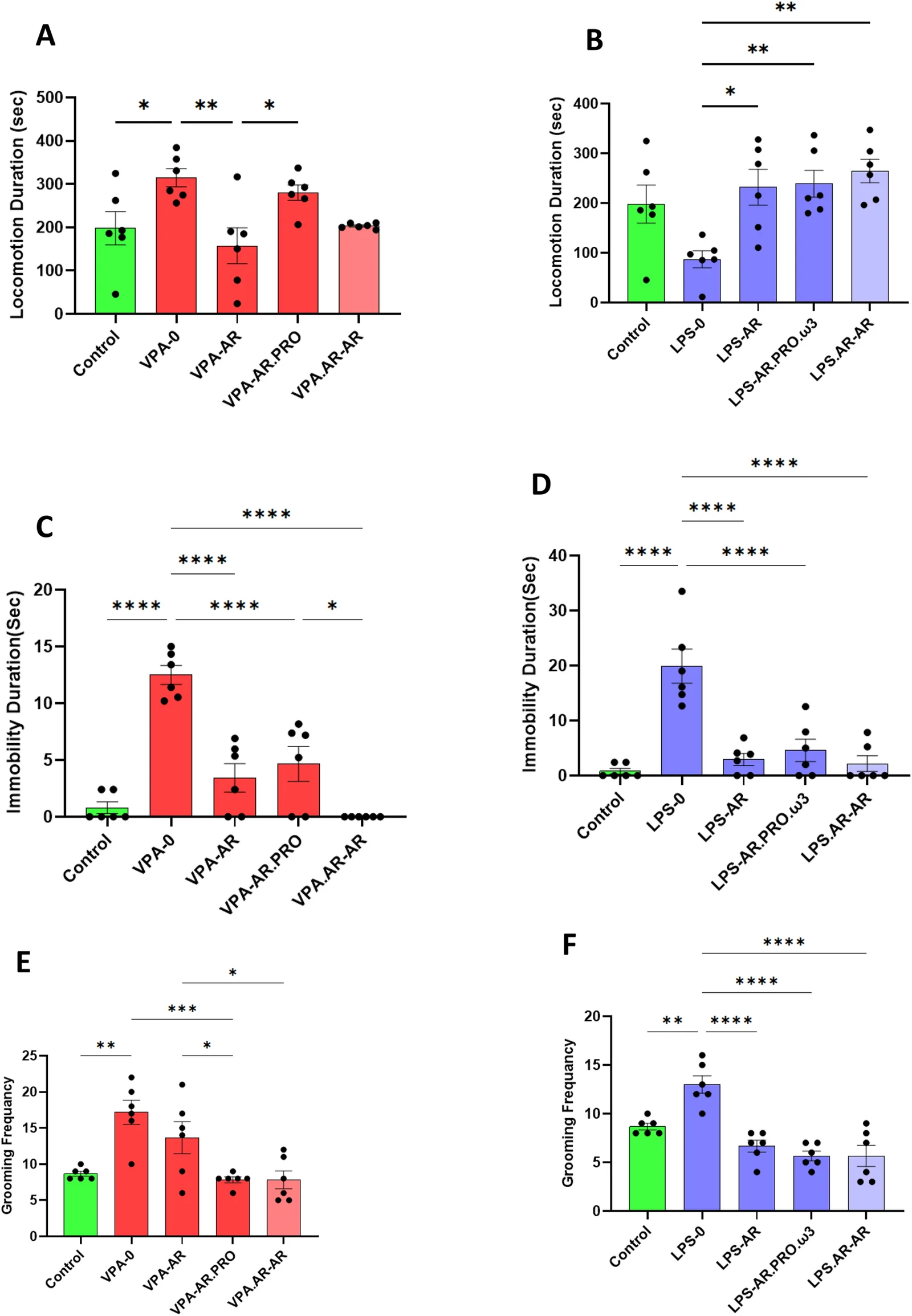
Locomotion total duration, immobility duration and grooming frequency in control and experimental groups. Saline (n=6), VPA (n=6; autism model), VPA-AR (n=6; artichoke treatment), VPA-AR.PRO (n=6; artichoke + probiotic), VPA-AR-AR (n=6; protective), LPS (n=6; autism model), LPS-AR (n=6; artichoke treatment), LPS-AR.PRO.ω3 (n=6; artichoke + probiotic + omega-3), and LPS-AR-AR (n=6; protective). Data are expressed as mean ± SEM. One-way ANOVA significance: ****p≤0.0001, ***p≤0.001, **p≤0.01, *p≤0.05). A, locomotion total duration of VPA and B, locomotion total duration of LPS; C, immobility total duration of VPA; D, immobility total duration of LPS; E, grooming frequency of VPA; F, grooming frequency of LPS; VPA, valproic acid; AR, artichoke; Pro, probiotics; ω3, omega3; LPS, lipopolysaccharide.

Behavioral data coding of the rats’ social behaviors was conducted using BORIS software, focusing on key parameters such as the duration and frequency of social interactions and percent social interaction per duration (Figure 2), time spent immobile and its frequency during the test as well as grooming behavior frequency and locomotion duration (Figure 3). As shown in Figure 2, social interactions within the social chamber were analyzed. Significant differences in time spent interacting with the novel rat were observed between the control, VPA, and LPS groups (F (4, 25) = 31.09, p<0.0001; Table 1, F (4, 25) = 13.59, p<0.0001; Table 2). Both the VPA-0 and LPS-0 groups exhibited significantly reduced social interaction compared to the control group (p≤0.01 for VPA-0 vs. control; p≤0.05 for LPS-0 vs. Control).

**Table 1:** Comparisons between the VPA groups in the following parameters.

Parameters	Groups	N	Mean ± S.E.	Median	F (DFn, DFd)	P value
Social interaction duration (sec)	Control	6	132.05 ± 27.76^**b**^	149.89	31.094 (4, 25)	<0.001
	VPA-0	6	34.71 ± 2.97^**a**^	35.19		
	VPA-AR	6	40.62 ± 18.19^**a**^	39.23		
	VPA-AR.PRO	6	151.29 ± 4.39^**b**^	150.91		
	VPA.AR-AR	6	255.93 ± 14.23^**c**^	248.65		
Social interaction frequency	Control	6	12.00 ± 2.44^**b**^	14.00	4.876 (4, 25)	0.005
	VPA-0	6	4.67 ± 0.21^**a**^	5.00		
	VPA-AR	6	5.83 ± 2.61^**ab**^	5.50		
	VPA-AR.PRO	6	12.00 ± 0.68^**b**^	12.50		
	VPA.AR-AR	6	11.83 ± 0.83^**b**^	12.00		
% time in social champer	Control	6	56.06 ± 13.50^**a**^	54.97	1.132 (4, 25)	0.364
	VPA-0	6	36.46 ± 6.89^**a**^	32.35		
	VPA-AR	6	38.90 ± 17.15^**a**^	26.86		
	VPA-AR.PRO	6	56.58 ± 4.11^**a**^	57.38		
	VPA.AR-AR	6	60.58 ± 3.04^**a**^	62.41		
Locomotion duration (sec)	Control	6	198.17 ± 38.35^**ab**^	189.60	5.304 (4, 25)	0.003
	VPA-0	6	314.85 ± 20.69^**c**^	307.90		
	VPA-AR	6	157.47 ± 41.40^**a**^	167.83		
	VPA-AR.PRO	6	280.42 ± 17.90^**bc**^	284.54		
	VPA.AR-AR	6	203.47 ± 2.51^**abc**^	202.96		
Immobility duration (sec)	Control	6	0.80 ± 0.51^**ab**^	0.00	25.471 (4, 25)	<0.001
	VPA-0	6	12.49 ± 0.83^**c**^	12.40		
	VPA-AR	6	3.44 ± 1.25^**ab**^	3.88		
	VPA-AR.PRO	6	4.66 ± 1.53^**b**^	6.21		
	VPA.AR-AR	6	0.00 ± 0.00^**a**^	0.00		
Grooming frequency	Control	6	8.67 ± 0.33^**ab**^	8.50	9.224 (4, 25)	<0.001
	VPA-0	6	17.17 ± 1.68^**c**^	17.50		
	VPA-AR	6	13.67 ± 2.22^**bc**^	14.50		
	VPA-AR.PRO	6	7.83 ± 0.40^**a**^	8.00		
	VPA.AR-AR	6	7.83 ± 1.25^**a**^	7.00		

This table describes One-way ANOVA Test between different groups for each parameter with Multiple Comparisons (Tukey test) within the entire groups for each parameter. The groups which have different letters are significant difference with each other at significant level (0.05). The groups which have the same letter are not significant with each other.

**Table 2:** Comparisons between the LPS groups in the following parameters.

Parameters	Groups	N	Mean ± S.E.	Median	F (DFn, DFd)	P value
Social interaction duration (sec)	Control	6	132.05 ± 27.76^**b**^	149.89	13.587 (4, 25)	<0.001
	LPS-0	6	50.22 ± 10.25^**a**^	57.04		
	LPS-AR	6	251.65 ± 23.77^**c**^	252.44		
	LPS-AR.PRO.ω3	6	120.21 ± 14.35^**ab**^	112.15		
	LPS.AR-AR	6	137.06 ± 16.81^**b**^	125.44		
Social interaction frequency	Control	6	12.00 ± 2.44^**b**^	14.00	4.396 (4, 25)	0.008
	LPS-0	6	5.33 ± 1.09^**a**^	6.00		
	LPS-AR	6	13.00 ± 0.97^**b**^	13.00		
	LPS-AR.PRO.ω3	6	10.33 ± 1.17^**ab**^	9.50		
	LPS.AR-AR	6	11.67 ± 0.99 ^**b**^	11.50		
% time in social champer	Control	6	56.06 ± 13.50^**a**^	54.97	1.286 (4, 25)	0.302
	LPS-0	6	54.46 ± 12.85^**a**^	50.24		
	LPS-AR	6	74.56 ± 8.99^**a**^	72.84		
	LPS-AR.PRO.ω3	6	46.75 ± 2.04^**a**^	48.31		
	LPS.AR-AR	6	65.73 ± 4.49^**a**^	60.74		
Locomotion duration (sec)	Control	6	198.17 ± 38.35^**ab**^	189.60	5.616 (4, 25)	0.002
	LPS-0	6	87.11 ± 16.92^**a**^	92.07		
	LPS-AR	6	231.81 ± 35.88^**b**^	246.92		
	LPS-AR.PRO.ω3	6	239.05 ± 26.75^**b**^	212.74		
	LPS.AR-AR	6	264.73 ± 23.57^**b**^	267.07		
Immobility duration (sec)	Control	6	0.80 ± 0.51^**a**^	0.00	17.836 (4, 25)	<0.001
	LPS-0	6	19.90 ± 3.11^**b**^	17.56		
	LPS-AR	6	2.92 ± 1.08 ^**a**^	3.28		
	LPS-AR.PRO.ω3	6	4.58 ± 2.03^**a**^	3.50		
	LPS.AR-AR	6	2.18 ± 1.42^**a**^	0.00		
Grooming frequency	Control	6	8.67 ± 0.33^**a**^	8.50	17.561 (4, 25)	<0.001
	LPS-0	6	13.00 ± 0.89^**b**^	12.50		
	LPS-AR	6	6.67 ± 0.62^**a**^	7.00		
	LPS-AR.PRO.ω3	6	5.67 ± 0.49^**a**^	5.50		
	LPS.AR-AR	6	5.67 ± 1.09^**a**^	5.50		

This table describes One-way ANOVA Test between different groups for each parameter with Multiple Comparisons (Tukey test) within the entire groups for each parameter. The groups which have different letters are significant difference with each other at significant level (0.05). The groups which have the same letter are not significant with each other.

Furthermore, the VPA-AR group showed a significant reduction in interaction time compared to the control group (p≤0.001), whereas the LPS-AR group demonstrated a significant increase in social interaction duration relative to the LPS-0 group (p≤0.0001). Notably, the LPS-AR group also exhibited significantly higher social interaction levels than the control group (p≤0.01).

The VPA-AR.PRO group showed a significant improvement in social interaction compared to both VPA-0 and VPA-AR (p≤0.001 for both comparisons). Among the VPA-treated groups, the protective group VPA-AR.AR showed the most substantial improvement, with interaction times significantly higher than VPA-0 (p≤0.0001), VPA-AR (p≤0.0001), and VPA-AR.PRO (p≤0.01). Similarly, the LPS-AR.AR protective group showed a significant increase in interaction time compared to LPS-0 (p≤0.05) were LPS-AR.PRO.ω3 does not show a significant difference with control or LPS-0 groups.

In terms of social interaction frequency (F (4, 25) = 4.876, p=0.0048; Table 1, F (4, 25) = 4.396, p=0.0079; Table 2), both VPA-0 and LPS-0 groups exhibited significantly reduced interaction frequency compared to the control group (p≤0.05 for both). The VPA-AR group does not show a significant difference with control or VPA-0 groups were VPA-AR.PRO group showed a significant increase in frequency relative to VPA-0 (p≤0.05). Additionally, VPA-AR.AR protective group demonstrated a significantly higher frequency of social interaction compared to VPA-0 (p<0.05). The LPS-AR group showed a significant increase in interaction frequency compared to LPS-0 (p≤0.05). While LPS-AR.PRO.ω3 and LPS-AR.AR group does not show a significant difference with control or LPS-0 groups. In addition, Figure 2 illustrates the percentage of time spent in the social chamber across the experimental groups. In panels C (F (4, 25) = 1.132, p=0.3642, Table 1) and F (F (4, 25) = 1.286, p=0.3023, Table 2) did not reveal statistically significant differences between groups, although a non-significant trend toward reduced social preference was observed in the VPA-exposed model.

Figure 3 illustrates the locomotion duration, duration of immobility and grooming frequency behaviors across the experimental groups. In panels A and B, of Figure 3, presents data on locomotion duration, The VPA-0 group (F (4, 25) = 5.304, p=0.0031, Table 1) shows a statistically significant hyperactivity (p≤0.05) in locomotion compared to the control. While LPS -0 group seems to cause hypoactivity. With artichoke treatment in VPA-AR group significantly drops (F (4, 25) = 5.616, p=0.0023, Table 2) locomotion (p≤0.01) compared to the VPA-0 group, while combination treatment with probiotics reduced hyperactivity compared to VPA-0, but remains notably more active than the control group. Meanwhile in the protected group (VPA-AR.AR) shows stable results, with locomotion levels almost matching the control. In the other hand, LPS-AR, LPS-AR.PRO.ω3, and LPS.AR-AR significantly increased locomotion (p≤0.05 and p≤0.01) compared to the LPS-0 group.

In panels C (F (4, 25) = 25.47, p<0.0001, Table 1) and D (F (4, 25) = 17.84, p<0.0001, Table 2) both the VPA-0 and LPS-0 groups exhibited significant increase in immobility duration compared to the control group (p≤0.0001). Treatment with artichoke extract (VPA-AR and LPS-AR) significantly reduced immobility duration compared to their respective untreated groups (p≤0.0001). Similarly, the VPA-AR.PRO and LPS-AR.PRO.ω3 groups also showed significantly reduced immobility compared to the VPA-0 and LPS-0 groups (p≤0.0001).

Notably, the protective groups (VPA-AR.AR and LPS-AR.AR) displayed near-complete normalization of activity, with immobility durations comparable to the control group and significantly lower than both VPA-0 and LPS-0 (p≤0.0001). Figure 3 also presents data on grooming behavior, including frequency. In panels E (F (4, 25) = 9.224, p=0.0001, Table 1) and F (F (4, 25) = 17.56, p<0.0001, Table 2), the VPA-0 and LPS-0 groups exhibited a significant increase in grooming frequency compared to the control group (p≤0.05), indicating elevated repetitive behavior.

The VPA-AR intervention significantly reduced grooming frequency compared to VPA-0 (p≤0.05), normalizing it to levels comparable to the control group. Both the VPA-AR.PRO and VPA-AR.AR groups further reduced grooming frequency beyond control levels (p≤0.001 vs. VPA-0). Similarly, the LPS-AR treatment significantly reduced grooming frequency compared to LPS-0 (p≤0.001). The LPS-AR.PRO.ω3 intervention normalized grooming frequency to control levels (p≤0.001 vs. LPS-0), while the protective group LPS-AR.AR reduced it even further – significantly below both the LPS-0 and control levels (p≤0.001).

## Discussion

The current findings demonstrate that both VPA and LPS prenatal exposures induce significant behavioral alterations characteristic of ASD, including reduced social interaction and increased repetitive behaviors (grooming) as well as anxiety-like behavior. Interventions with Artichoke (as a prebiotic), alone or in combination with probiotics and/or omega-3, consistently ameliorated these behavioral phenotypes.

Starting with social interaction duration and frequency (Figure 2A, B, C), the VPA-exposed group (VPA-0) showed a marked impairment in social interaction and shows a noticeable drop in the mean time spent in the social chamber, confirming the toxic effect of *in utero* VPA exposure, Similarly, Figure 3 (A, C, E) reveal a significant increase in locomotion activity, immobility and repetitive grooming behaviors, respectively, further demonstrating behavioral alterations relevant to ASD.

These results are in line with previous reports [7], 8], 35], 44], 45], which have consistently shown that prenatal exposure to VPA produces behavioral deficits resembling those observed in individuals with ASD and animal models, including pronounced social impairments and stereotyped behaviors.

Several mechanisms have been proposed to explain the antiseizure effects of VPA, including the enhancement of the inhibitory neurotransmitter γ-aminobutyric acid (GABA) in the brain, and the modulation of sodium and calcium ion channel influx and efflux. In addition, VPA acts as a histone deacetylase (HDAC) inhibitor, thereby inducing epigenetic modifications that alter cell proliferation and differentiation in developing tissues, including the fetal nervous system [46], 47]. Importantly, VPA can cross the placental barrier and enter fetal circulation, with fetal concentrations typically ranging from 70 to 100 % of maternal levels. Moreover, VPA is excreted into breast milk at concentrations comparable to plasma levels [8], 48], raising concerns about its teratogenic potential and developmental risks.

Additionally, offspring from mothers exposed to maternal immune activation (MIA) induced by LPS (LPS-0) showed significantly reduced social interaction duration and frequency (Figure 2 D, E), indicating that maternal infection during early gestation could be a risk factor for ASD. While chamber occupancy reflects spatial preference for social proximity, direct investigation measures provide a more specific index of active social engagement with the conspecific [49]. The percentage of time spent in the social chamber relative to the total test duration did not reveal statistically significant differences between groups, although a non-significant trend toward reduced social preference was observed in the VPA-exposed animals (Figure 2C). This apparent discrepancy between normalized chamber occupancy and direct social interaction measures may be explained by differences in general activity levels and exploratory behavior. Indeed, VPA and LPS exposed animals exhibited altered locomotor activity [50], [51], [52].

Likewise, Figure 3 (B, D, F) show hypo locomotion activity, increased immobility and repetitive grooming behaviors, further demonstrating ASD-relevant phenotypes. These findings are consistent with previous studies [13], 14], 20], 38] which report that prenatal LPS-induced MIA initiates neuroinflammatory processes in the developing fetal brain. Elevated levels of proinflammatory cytokines (TNF-α, IL-1β, IL-6, and IL-17) during gestation are strongly associated with behavioral impairments (social deficits, repetitive behaviors, and cognitive inflexibility in offspring) and hypomyelination, thereby providing a mechanistic link between maternal infection, neuroinflammation, and ASD-like outcomes.

Embryonic exposure to VPA or LPS offspring exhibited an increased immobility time compared to the control as mentioned in Figure 3 C, D, which might indicate an increased anxiety-like behavior, which is a behavioral feature of ASD models [53], 54]. However, validated tests of anxiety need to be performed in future studies to better assess anxiety-like behavior in this model of autism.

The therapeutic efficacy of *Cynara scolymus* (Artichoke) has been recognized since antiquity and is primarily attributed to its rich content of bioactive compounds, particularly polyphenols (e.g., caffeoylquinic acid derivatives such as chlorogenic acid, cynarin, neochlorogenic acid, and cryptochlorogenic acid) and flavonoids (e.g., luteolin, apigenin, kaempferol) [27], 55], 56]. Additionally, Artichoke is considered a prebiotic due to its high inulin content. Inulin, a soluble dietary fiber, undergoes fermentation by gut microbiota, promoting the growth of beneficial bacteria and thereby supporting gut health [26], 27].

Behavioral analyses revealed that artichoke alone (VPA-AR) did not significantly improve VPA-induced deficits in social interaction duration and frequency (Figure 2 A, B), nor in repetitive grooming behavior frequency (Figure 3E). However, it did ameliorate immobility and locomotion activity (Figure 3A, C). In contrast, the combination of artichoke as a prebiotic with probiotics (VPA-AR.PRO) significantly ameliorated social interaction deficits and repetitive behaviors induced by VPA, as shown in Figures 2 (A, C) and 3 (A, C, E). These results suggest that Artichoke’s modulatory effects may be enhanced through synergistic interactions with probiotics.

Mechanistically, VPA exposure during pregnancy is known to induce profound alterations in the gut, including inflammation and microbiota dysbiosis, thereby disrupting the gut–brain axis and influencing ASD-related behaviors [57], [58], [59]. The observed rescue effects of Artichoke–probiotic combinations support the growing evidence for targeting the microbiota–gut–brain axis as a therapeutic strategy for ASD.

In 2024, Prince and colleagues provided strong evidence that a prebiotic diet consisting of 3 % galacto-oligosaccharide/fructo-oligosaccharide (GOS/FOS; 9:1) normalized both immune and behavioral deficits in a VPA-induced mouse model of ASD. In contrast, the current investigation found that Artichoke alone had no corrective benefits as a prebiotic in improving VPA-induced social interaction deficiencies. This disparity could be related to changes in dosage (400 mg/kg in the current investigation), duration of treatment (23 days beginning on postnatal day 7), or timing of intervention as compared to Prince et al. who began food supplementation at birth and continued for 49 days [60].

On the other hand, previous research has demonstrated that combined probiotic and prebiotic interventions can alleviate a broad range of autistic-like symptoms in prenatal VPA-induced rodent models. These therapeutic effects appear to be mediated not only by behavioral improvements but also by modulation of inflammatory responses (e.g., IL-6, IL-10), neurotransmitter systems (serotonin, GABA), and restoration of gut microbiota balance [33], 61], 62]. Collectively, these findings support the use of synbiotic interventions as a promising therapeutic strategy for subsets of individuals with autism, acting through immune regulation, neurotransmitter modulation, and microbiota–gut–brain axis restoration.

As highlighted in previous studies, ASD-like behaviors are strongly associated with gut dysbiosis, which can increase intestinal permeability and allow the translocation of LPS into the bloodstream. Circulating LPS can activate immune responses within the brain, thereby contributing to neuroinflammation and the behavioral manifestations of ASD [7], 15], 38], 63]. In the present study, offspring from MIA induced by LPS that received Artichoke as a prebiotic alone (LPS-AR) or in combination with probiotics and omega-3 fatty acids (LPS-AR.PRO.ω3) exhibited remarkable improvements in social interaction behavior (Figure 2D, E, F) as well as reductions in immobility and repetitive behaviors (Figure 3D, F) also significantly increase in activity (Figure 3B). These findings suggest that prebiotics, probiotics, and omega-3 fatty acids mitigate the detrimental effects of prenatal LPS exposure and inflammation by modulating the gut microbiota and immune responses.

Mechanistically, probiotics and omega-3s reduce gut permeability, thereby lowering systemic LPS translocation and subsequent neuroinflammation, while prebiotics promote the growth of beneficial bacteria that further suppress LPS production. Through these microbiota-mediated processes, such interventions influence brain function and alleviate ASD-related behaviors [19], 22], 24], 25], 64], 65]. The present findings are in line with this evidence, further supporting the gut–brain axis as a critical therapeutic target.

The corrective role of omega-3 supplementation in MIA offspring was specifically evaluated in this study to clarify its contribution as part of a combined intervention with prebiotics and probiotics. Leyrolle et al. reported that omega-3s, together with prebiotics and probiotics, can positively modulate the gut–brain axis and mitigate MIA as risk of ASD behavioral features in rat offspring [66]. Thus, synergistic effects have been reported when omega-3s are combined with prebiotics and probiotics, with greater efficacy than single interventions alone. Such combinations have been shown to reduce inflammation, improve gut barrier integrity, and enhance neurodevelopmental outcomes in animal models [65], 67], 68]. These findings correlate with the present results, in which the combined intervention significantly improved social behaviors in the LPS-induced model of ASD.

Because MIA during pregnancy is strongly associated with an increased risk of ASD, it is of particular interest to evaluate the potential protective role of Artichoke supplementation during gestation in preventing the development of ASD-like behaviors in offspring [12], 20]. In the present study, dams administered artichoke as a prebiotic from the first day of gestation (protective dose) and offspring received postnatal therapeutic doses. As shown in Figures 2 and 3, this protective intervention alleviated ASD-related behavioral abnormalities. These findings suggest that protection against VPA-induced toxicity during pregnancy is more readily achieved compared with LPS-induced MIA. This highlights the fact that MIA represents a stronger risk factor for ASD, as maternal protection during gestation is less effective against immune-mediated toxicity. Mechanically, while both VPA and LPS are powerful preclinical models of ASD, the direct teratogenic effect of VPA is more amenable to prevention than the complex, multifactorial immune-inflammatory processes triggered by LPS [8], [11], [12], [13].

In summary, both VPA and LPS are widely used to model ASD, through either direct chemical toxicity or indirect immune-mediated mechanisms, respectively. Although both models result in overlapping behavioral and neurobiological phenotypes, their mechanistic differences shape distinct therapeutic challenges. Artichoke supplementation as prebiotic alone shows promise in mitigating grooming and immobility behavioral deficits induced by both VPA and LPS. However, the addition of probiotics enhances the therapeutic efficacy of Artichoke in the VPA model, particularly in restoring social interaction and reducing repetitive behaviors. In contrast, in the LPS model, the combination of Artichoke, probiotics, and omega-3 fatty acids effectively reduced immobility and normalized grooming behaviors but did not enhance social interaction beyond the effect of Artichoke alone. This suggests a complex interaction between dietary interventions and the immune-mediated pathways underlying MIA, indicating that tailored strategies may be required depending on the etiology of ASD.

## Conclusions

This study shows evidence of the pre/postnatal therapeutic role of artichoke as prebiotic in improving social impaired behavior in MIA by LPS and VPA-induced rodent models with persistent autistic features. The mechanism of action underlying the therapeutic effects of artichoke as prebiotic should be investigated in the near future. Studies of the protective effects of artichoke are also recommended. A key limitation of this study is the use of only male animals, based on the higher prevalence of ASD in males. This may limit detection of sex-related differences. Including both sexes in future work would provide a more complete understanding of potential sex-dependent mechanisms.
